# Adolescent Acceptability of School and Home Micronutrient Supplementation and Nutrition Curriculum in Mozambique

**DOI:** 10.1111/mcn.70211

**Published:** 2026-06-19

**Authors:** Sarah Bauler, Christine Marie George, Elli Leontsini, Mateus Mandlate, Joel Gittelsohn, Jamie Perin, Titus Kirwa, Carmen Tse, Tui Bernardo, Tom Davis, Asrat Dibaba Tolossa, Antonio Santana Dias, Julianne Wong, Lucilia Moises

**Affiliations:** ^1^ World Vision International London England UK; ^2^ Johns Hopkins Bloomberg School of Public Health Baltimore Maryland USA; ^3^ World Vision Mozambique Monapo Nampula Province Mozambique; ^4^ Global Health Consultant Torrevieja Alicante Spain; ^5^ World Vision Canada Toronto Ontario Canada

**Keywords:** acceptability, adolescent anemia, Mozambique, multiple micronutrient supplementation, nutrition curriculum, supplement

## Abstract

Iron‐deficiency anemia is the most common micronutrient deficiency and a leading cause of disability‐adjusted life years among adolescent girls and young women (AGYW) globally. Although multiple micronutrient supplementation (MMS) provides a broader range of micronutrients than iron‐folic acid supplementation (IFAS) prior to conception, the acceptability of MMS and home‐based supplementation strategies remains underexplored. We assessed the acceptability of MMS, IFAS, and a contextualized nutrition curriculum delivered via school clubs in a two‐arm cluster‐randomized trial across three rural secondary schools in Monapo District, Mozambique. Fourteen teachers (clusters) were randomized to deliver either weekly school‐based IFAS or daily home‐based MMS. A total of 492 AGYW aged 13–20 years were enrolled (240 IFAS; 252 MMS); both arms received the same nutrition curriculum. Participants in both arms reported increased energy, improved appetite, and relief from menstrual symptoms. IFAS was significantly more acceptable than MMS for smell, and some participants perceived the once‐weekly IFAS regimen as less burdensome than daily MMS. Some AGYW also reported that male peers perceived MMS as birth control, or assumed the girls were pregnant, due to the image of a pregnant woman on the pill bottle. Ratings of the nutrition curriculum and teachers' facilitation were positive in both arms. Participants generally preferred the regimen they were assigned, and family support facilitated adherence. These findings suggest home‐based supplementation may be a feasible and acceptable strategy for reaching in‐school and out‐of‐school AGYW in Mozambique. Including boys in future interventions and redesigning the MMS label could help reduce misconceptions and enhance acceptability.

## Summary

- Multiple micronutrient supplementation (MMS) and iron‐folic acid supplementation (IFAS) were both acceptable among adolescent girls in rural Mozambique.

- Social norms and gendered misconceptions, particularly related to supplement labeling, influenced acceptability and highlight the importance of adolescent‐centered design.

- Engaging boys and addressing community misperceptions may improve social acceptability of adolescent supplementation programs.

- Home‐based MMS delivery may offer an equitable strategy for reaching out‐of‐school girls in fragile contexts with high dropout rates.

## 1 Introduction

Globally, iron‐deficiency anemia (IDA) is the most common micronutrient deficiency and a leading cause of disability‐adjusted life years (DALYs) lost among adolescent girls and young women (AGYW) aged 10–19 years (Canavan and Fawzi 2019). Heavy menstrual bleeding can exacerbate IDA and is often an underemphasized issue among adolescent girls (Cooke et al. 2017). Malnutrition, particularly IDA, undermines cognitive ability and classroom performance, income potential, and maternal and infant health outcomes (Best et al. 2010). In Mozambique, 54.9% of girls between 15 and 19 years of age are estimated to have IDA (World Health Organization 2018), and the average age of first pregnancy is 19 years (World Population Review 2025). Adolescent girls and young women with anemia are at a higher risk for multiple adverse pregnancy outcomes, including preterm birth, low birth weight, and postpartum hemorrhage (Wang et al. 2025).

School‐based iron‐folic acid supplementation (IFAS) programs have been implemented in low‐ and middle‐income countries, including Mozambique, as a strategy to build iron stores before pregnancy and prevent IDA among menstruating AGYW (Cliffer et al. 2023). However, strategies for delivering supplementation to adolescents at home or out of school remain underexplored (Healthy Mothers Healthy Babies Consortium 2025). Emerging evidence indicates that multiple micronutrient supplementation (MMS) during pregnancy is superior compared to IFAS in reducing the risk for low birthweight babies (−19% reduction) and infant mortality at 6 months (−29% reduction) (Smith et al. 2017), and its broader nutrient profile may offer additional benefits for adolescent girls who face multiple micronutrient deficiencies beyond iron and folate alone (Cliffer et al. 2023). If Mozambique transitioned from weekly IFAS to daily MMS, an estimated 384,443 DALYs could be averted (Nutrition International 2020). Thus, MMS presents a promising approach to integrate with adolescent nutrition education to decrease IDA among AGYW. While Mozambique has an established national nutrition curriculum for primary school‐aged children, this curriculum does not extend to secondary schools and does not include structured adolescent‐focused nutrition education or guidance on micronutrient supplementation. As a result, older adolescent girls—who have heightened iron needs and high anemia prevalence—lack access to standardized school‐based nutrition programming and are not systematically reached with anemia prevention messaging aligned with supplementation strategies. (Homiak 2016). Furthermore, although over half of girls drop out of school before fifth grade (USAID Advancing Girls' Education 2023), no national strategy exists to reach girls not in school with micronutrient supplementation.

In collaboration with the Mozambique Ministries of Health, Education, and Culture, World Vision Mozambique implemented a two‐arm cluster‐randomized trial complemented by a mixed‐methods process evaluation to evaluate the acceptability, adherence and effectiveness of two adolescent micronutrient supplementation strategies, daily home‐based MMS and weekly school‐based IFAS, delivered alongside a contextualized nutrition education curriculum. This manuscript focuses on examining the acceptability and adherence of the interventions among AGYW and their teachers, while effectiveness outcomes are reported separately, pending publication (Bauler et al. 2025).

## 2 Methods

### 2.1 Study Design and Setting

This study was conducted from July to October 2024 as part of a two‐arm cluster‐randomized trial with an embedded mixed‐methods process evaluation across three rural secondary schools in Monapo District, Nampula Province, Mozambique. The process evaluation consisted of a pre‐ and post‐intervention survey complemented by a qualitative inquiry. Three schools were purposively selected within the largest operational district of the *Every Girl Can* initiative, a gender‐transformative initiative aimed at promoting girls' safety, empowerment, and equity, to align with the broader programmatic context and logistical feasibility. All three schools served students from remote, low‐resource communities. Two were situated near a paved road, whereas the third was located approximately 10 km away. Regardless of location, many students walked two to 3 h each day to attend school.

A total of 492 adolescent girls aged 13–20 years were enrolled in the study (240 in the IFAS arm and 252 in the MMS arm). Girls participated through school‐based clubs, which served as the primary platform for delivering the nutrition curriculum and supplementation interventions. Club size was determined by class enrollment and ranged from approximately 13–25 girls per club. In total, 28 clubs were formed across the three schools. Teachers were eligible to participate if they were teaching at one of the three participating secondary schools and agreed to serve as facilitators for the girls' clubs and nutrition curriculum. Teachers (*n* = 14) served as the unit of randomization, with each teacher assigned to facilitate two clubs, resulting in 14 clusters. Table 1 presents the distribution of clusters, participants, and age groups across the three school.

**Table 1:** Cluster randomization and participant distribution.

Cluster	Intervention Arm	School	Participants (n)	Age Distribution
Teacher 1	IFAS	School 1	49	13 to 16 years
Teacher 2	MMS	School 1	47	13 to 16 years
Teacher 3	MMS	School 1	48	17 to 20 years
Teacher 4	MMS	School 2	35	13 to 20 years
Teacher 5	IFAS	School 2	39	13 to 20 years
Teacher 6	IFAS	School 3	30	13 to 16 years
Teacher 7	MMS	School 3	30	13 to 16 years
Teacher 8	MMS	School 3	30	13 to 16 years
Teacher 9	MMS	School 3	30	13 to 20 years
Teacher 10	IFAS	School 3	33	13 to 20 years
Teacher 11	IFAS	School 3	28	17 to 20 years
Teacher 12	IFAS	School 3	32	16 to 20 years
Teacher 13	IFAS	School 3	29	13 to 20 years
Teacher 14	MMS	School 3	32	17 to 20 years
**Total Overall**		**3 schools**	**492**	

To tailor the learning experience, clubs were stratified by age group (13–16 years and 17–20 years), although a few clubs included participants outside these ranges.

Teachers were randomly assigned to either the IFAS or MMS arm using a stratified and constrained randomization approach (Yu et al. 2019). Prior to implementation, teachers completed a 2‐day in‐person training led by the Study Coordinator and Nutrition Officer on curriculum content and facilitation, including role‐play exercises. Teachers were provided with standardized facilitator guides and received ongoing supervisory support from the Study Coordinator throughout the 5‐month implementation period, including guidance on supplement distribution and adherence monitoring. Each teacher received a small monthly stipend ($20 USD) as an incentive. They also signed commitment forms outlining their responsibilities, including facilitating weekly nutrition curriculum sessions, tracking attendance, engaging with participants, and reporting any issues to the Study Coordinator.

In the IFAS arm, teachers distributed iron‐folic acid supplements (containing 90 mg of elemental iron and 400 µg of folic acid) once a week for 5 months during club sessions and directly observed consumption. In the MMS arm, participants received a 180‐count bottle of MMS to take daily at home for 5 months. Each MMS tablet contained 30 mg of iron, 400 µg of folic acid, 800 µg of vitamin A, 70 mg of vitamin C, and recommended amounts of vitamins D, E, B1, B2, B6, B12, niacin, zinc, iodine, selenium, and copper, and was procured through Vitamin Angels in partnership with Kirk Humanitarian (Kirk Humanitarian 2025). MMS participants also received a tracking sheet and an information leaflet, which they reviewed together with teachers. The leaflet covered daily use, safe storage, and clarified that MMS did not prevent pregnancy. MMS tracking sheets were collected at the endline. Both IFAS and MMS adhered to WHO and UNIMMAP dosing guidelines (Kirk Humanitarian 2025). Participants were monitored for adverse events throughout the study period.

Both arms received the same contextualized nutrition education curriculum, which was co‐designed with adolescent participants through photovoice and adolescent‐centered design workshops (Bauler et al. 2025). Teachers facilitated weekly, 1‐h club sessions drawing from five modules, held during early morning or lunchtime. Curriculum topics included nutrition goals and habits, understanding anemia, gender and community relationships, peaceful conflict resolution, and service learning. Practical sessions included cooking demonstrations using locally available iron‐rich foods and encouraged intergenerational knowledge‐sharing. Both arms also received messages on the benefits of supplementation, with the MMS arm additionally receiving information on safe storage of MMS bottles and strategies to support daily adherence. The final module involved a community service project designed and implemented by students in collaboration with school and community leaders, reinforcing learning and building agency (World Vision International 2025). Project suggestions included establishing a school garden, constructing a nutrition classroom, and building latrines to improve sanitation, and project selection varied by school.

The trial is registered at ClinicalTrials.gov (NCT06815315), and impact findings on anemia outcomes are pending publication (Bauler et al. 2025).

### 2.2 Ethics Statement

Ethical approval for the human subject research was obtained from the Mozambique National Committee of Bioethics for Health (IRB00002657). The Johns Hopkins Bloomberg School of Public Health IRB (FWA #00000287) determined this activity to be public health practice, as defined by United States Department of Health and Human Services regulations at 45 CFR 46.102. Although the Mozambican National Committee of Bioethics for Health does not require caregiver consent for adolescents aged 18 and above, we obtained consent from all participants and their caregivers regardless of age.

### 2.3 Data Collection

Although all girls' club participants received the interventions, we required a sub‐sample of 280 to investigate the effects of supplementation on anemia reduction. The detailed methodology, study flow chart, and rationale for this sample size determination are elaborated in Bauler et al. submitted for publication (Bauler et al. 2025). To randomly select participants for a cross‐sectional baseline survey, each girl from her respective club drew a number from a basket. Girls who selected numbers 1 through 10 were included in the survey sample, resulting in 10 randomly selected participants from each club, for a total sample of 280 girls.

We piloted, refined, and conducted the baseline survey over 7 days from April 9 to 16, 2024. Six female enumerators were trained to administer the survey, recognizing that some questions were sensitive and that adolescent girls might feel more comfortable discussing them with female interviewers. The survey instrument collected data on demographics, 24‐h dietary recall, food security, and the type of fuel most often used for cooking. Interviews took place in private settings at school. Built‐in validation checks ensured that enumerators did not enter invalid or out‐of‐range responses and enforced skip logic.

After 5 months of supplement and curriculum implementation, we repeated the survey among the same girls using the same enumerators and health technicians, between September 18 and 24, 2024. We added questions to the endline survey to assess the acceptability and adherence of IFAS and MMS among the girls, focusing on preferences, ease of use, support systems, and potential barriers. Key variables included preferred timing and location for supplement consumption, ease of remembering and swallowing supplements, perceived side effects, perceived benefits, palatability of supplements, and supportive behaviors from family members (e.g., reminders, encouragement) and teachers (e.g., supervision during intake, reinforcement during club sessions). A total of 280 adolescent girls completed the endline survey and were included in the analytic sample for the acceptability‐related variables.

In the qualitative inquiry, we explored acceptability and adherence further through focus group discussions (FGDs). We purposively selected 10 girls from each school—five from the MMS arm and 5 from the IFAS arm, representing all grades 7 through 12 —and conducted three FGDs, one at each school. Sample sizes for girls and teachers were guided by anticipated thematic saturation (Weller et. al. 2018) and by information power aligned with our study aims (Malterud et al. 2016).

Teachers assisted in identifying eligible participants, who were invited to participate voluntarily and provided informed consent prior to the discussions. We invited all 14 teachers to participate in three discussions, one discussion per school. Nine teachers ultimately participated in the discussions: four at one school, three at a second, and two at the third.

The FGD and teacher discussion guides were informed by individual‐level constructs in the theoretical framework of acceptability (TFA) (Sekhon et al. 2017) and by broader social and structural influences through the lens of the social and ecological model (SEM) (McLeroy et al. 1988), while enabling emerging findings grounded in the participants' empirical feedback (Ryan and Bernard 2003). The FGD Guide explored participants' perceptions of the interventions delivered, including aspects they found most beneficial or challenging, key learnings, and suggested improvements. Discussions about the nutrition curriculum examined the acceptability of service projects, cooking demonstrations, and club meetings, and the role of teachers as facilitators. Participants were also asked to reflect on their adherence, including any social support received, perceived benefits, challenges, side effects, and supplement preferences. The teacher discussion guide assessed educators' perspectives on the implementation of the nutrition curriculum, including the ease of teaching, lesson effectiveness, and observed program benefits, with feedback on service projects, cooking demonstrations, and student engagement. Discussions also explored support for girls' participation and comparative feedback on MMS versus IFAS, focusing on acceptability and perceived effectiveness.

The FGDs and teacher discussion groups were facilitated by the Study Coordinator, with note‐taking conducted by the project Monitoring and Evaluation Officer. Both were trained on the protocol for informed consent, guidance on administering open‐ended and non‐leading questions, probing techniques, group dynamics, reflexivity, and field note‐taking procedures. Before starting discussions, the facilitator read the consent script to confirm participants' consent and seek permission to record the discussion, assuring that no names would be transcribed to ensure confidentiality. FGDs and teacher discussion groups were audio‐recorded, with participants' consent, transcribed, and translated into English from Portuguese for analysis.

### 2.4 Data Analysis

The quantitative data (i.e., baseline participant characteristics, variables assessed at baseline and follow‐up) were analyzed descriptively. Acceptability variables were assessed at endline. Statistical significance of differences between intervention arms were assessed using chi‐square tests, Fisher's exact test, and *t*‐tests, and analysis was conducted in R (R Core Team 2025).

**Figure 1:**
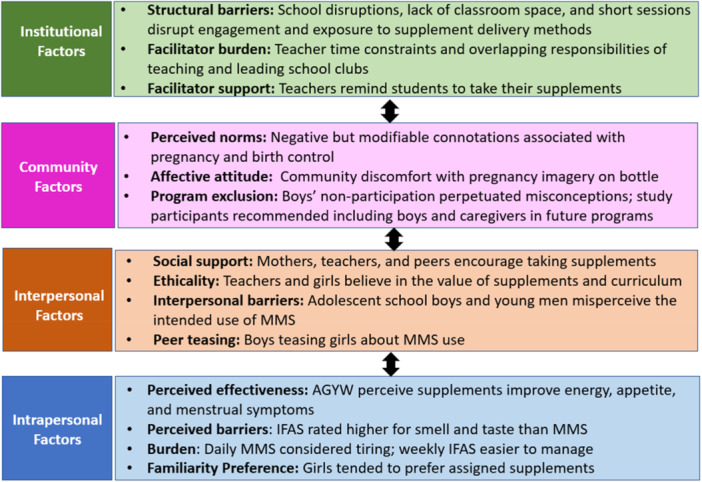
Emergent model derived from the study findings, informed by the TFA and SEM, illustrating how intrapersonal, interpersonal, community, and institutional‐level factors influence different dimensions of acceptability of adolescent micronutrient supplementation in rural Mozambique.

We defined acceptability as the extent to which the intervention is perceived as appropriate, desirable, and feasible by both its intended users and the implementers. Qualitative data analysis was guided by theoretical frameworks: the TFA to examine individual‐level dimensions (affective attitude, burden, perceived effectiveness, and ethicality) (Sekhon et al. 2017); and the social ecological model (SEM) to capture broader social and structural influences, including social acceptability (peer and community perceptions), cultural relevance, and logistical feasibility (McLeroy et al. 1988). Results were synthesized into an emergent model to visually summarize multi‐level influences on acceptability and adherence, shown in Figure 1.

We employed both deductive and inductive reasoning to develop a codebook for the qualitative analysis (Elo and Kyngäs 2008). The SEM and TFA informed the initial, deductive codes and codebook structure, while inductive coding involved identifying and adding new codes and subcodes that emerged from the transcripts (Ryan and Bernard 2003). The PI developed the initial codebook: two master's‐level interns, trained in MAXQDA, then independently double‐coded the same transcripts using the codebook. After coding, the PI met with the coders to review coded segment reports, revise the codebook, and add new codes that emerged inductively from the data. The PI led team discussions to resolve coding disagreements and made final coding decisions. Horizontal analysis was used to identify emerging themes across FGDs and teacher discussion transcripts, while vertical analysis focused on an in‐depth understanding of an identified theme within a transcript (Gaudet and Robert 2018). We judged a theme based on its relevance to the study aims, recurrence and saturation across teacher and adolescent transcripts, the richness/intensity of the account, and its ability to illuminate or challenge relevant theoretical constructs. Coded segment reports were generated to systematically organize and categorize common themes and sub‐themes. Qualitative data were managed and coded using MAXQDA (18). Qualitative findings were triangulated with quantitative results (Carter et al. (2014)), with each analyzed separately and then integrated by iteratively comparing findings and mapping key themes to components of the emergent model. While the study's findings were rooted in the specific context of Mozambique, our analysis and its documentation aimed to facilitate transferability, allowing readers to assess the relevance of these results to their own contexts (Graneheim and Lundman 2004).

## 3 Results

### 3.1 Cluster Randomization and Participant Characteristics

Of the 280 participants selected for the survey, baseline data were available for 277 participants (145 IFAS; 132 MMS), as three participants were not found in the study registry at baseline. Most participants were over the age of 14 (76%, 210/277) and lived in households with more than five members (58%, 161/277). Thirty‐nine percent (108/277) of participants came from households with fathers who had no formal education, and 22% (61/277) had fathers who had completed only primary school. The majority of fathers (80%, 223/277) were engaged in farming. Nearly all households (99%, 276/277) relied on solid cooking fuels (coal and firewood). The most common household water source was piped water to the neighborhood (67%, 185/277), followed by hand‐dug wells or surface water (24%, 65/277), and piped water directly to the home (10%, 27/277). Pit latrines were the most commonly used sanitation facility (92%, 255/277).

At baseline, 83% (230/277) of participants had already experienced menarche. Recent illness symptoms were common: 53% (148/277) reported a cough in the past month, and 41% (115/277) reported a malaria diagnosis during the same period. Food insecurity was prevalent, with 72% (198/277) of participants reporting going to bed hungry at least once in the past 30 days. Among those who went to bed hungry, 93% (184/198) reported it occurring sometimes (3–10 times), while 7% (14/198) reported it happening rarely (1–2 times). Additionally, 22% (62/277) of respondents reported going an entire day or night without eating in the past month.

Menarche status differed significantly between arms (*p* = 0.03), with a higher proportion of girls in the IFAS arm (88%, 128/145) reporting that they had reached menarche compared to those in the MMS arm (77%, 102/132). All other baseline characteristics were balanced, with no statistically significant differences observed between the study arms.

### 3.2 Intrapersonal Level Perceptions: Perceived Effectiveness and Burden

Endline survey data were available for 273 participants, as five participants were not found in the study registry at endline. We assessed participants' perceptions of acceptability by asking whether they preferred IFAS (weekly, consumed at school) or MMS (daily, consumed at home), as girls in the IFAS group were aware of the MMS regimen used by their peers in the MMS group within the same school, and vice versa. As shown in Table 2, in the IFAS arm, 86% preferred IFAS, while 14% preferred MMS and 3% reported no preference. In the MMS arm, 72% preferred MMS, while 28% preferred IFAS. Although the preference for IFAS was slightly higher than for MMS, these findings suggest that participants generally preferred the supplement regimen to which they were assigned and had positive attitudes towards both formulations. A sensitivity analysis stratified by menarche status showed similar patterns of supplement preference across groups, suggesting that the baseline imbalance in menarche status did not meaningfully influence the acceptability findings.

**Table 2:** Supplement preference among both arms.

Intervention arm	Prefer IFAS (%) (*n*)	Prefer MMS (%) (*n*)	p‐value¹
IFAS Arm (*n* = 135)	86% (116)	14% (19)	< 0.001
MMS Arm (*n* = 131)	27% (35)	73% (96)	< 0.001

¹p‐values from the chi‐square test of independence to compare differences in supplement preference between intervention arms. Percentages are based on available responses; totals are less than the full sample due to missing or “don't know” responses.

Four prominent themes emerged from the FGDs regarding the perceived effectiveness of micronutrient supplements: (1) increased energy and physical resilience; (2) enhanced appetite and nutritional intake; (3) menstrual symptom relief, and (4) an overall sense of well‐being. Several participants from both arms reported feeling stronger, less fatigue throughout the day, and having greater endurance for daily activities after starting IFAS and MMS supplementation. A few participants also mentioned feeling an improvement in immunity against illness and colds. A number of participants, in both intervention arms, reported a noticeable increase in appetite, which led to higher food consumption. One participant shared,

They've (the supplements) done me a lot of good; they've given me energy, and I've noticed that I feel more like eating now. I didn't eat much before, but now I feel more appetite and it's helped me.

(IFAS supplement recipient, Participant 3, School 1 FGD; Intrapersonal level: perceived effectiveness)

Several participants also shared that consuming the micronutrient supplements reduced menstrual discomfort and fatigue, with some noting they felt less weak and dizzy during menstruation. Menstrual symptom relief was also linked to improvements in school attendance, with one participant noting,

In the past, girls used to drop out of school quickly, but now they don't. Also, there are girls who get very thin when they menstruate, so those pills helped us a lot because they reduced our pain and (helped us) recover a lot.

(IFAS supplement recipient, Participant 3, School 3 FGD; Intrapersonal level: perceived effectiveness)

Overall, participants in both arms widely described feeling a general improvement in their physical and emotional health with supplement use, most often described in the FGDs as “good in their bodies.” Both teachers and girls in their respective teacher discussion groups and FGDs noted that improved wellbeing led to greater ability to focus in class and more engagement in learning.

The qualitative findings are supported by the quantitative data, as shown in Table 3. A Likert scale (strongly agree to strongly disagree) was used to assess the perceived effectiveness of IFAS and MMS across four domains: feeling healthier and happier, improved appetite, feeling more energetic, and experiencing no stomach upset or constipation. Overall, perceived effectiveness was generally high in both groups, but was slightly higher for IFAS compared to MMS. However, these differences were not statistically significant.

**Table 3:** Perceived effectiveness of IFAS compared to MMS (agree and strongly agree responses only).

Acceptability Indicator	IFAS (%) (*n*)	MMS (%) (*n*)	p‐value¹
Healthier and happier	93% (130)	90% (123)	0.36
More energy	88% (123)	86% (118)	0.67
Improved appetite	86% (120)	87% (119)	0.79
No stomach upset or constipation	66% (93)	66% (91)	1.00

¹p‐values derived from chi‐square tests comparing perceived effectiveness between IFAS and MMS groups across four acceptability domains. Percentages are based on available responses; totals are less than the full sample due to missing or “don't know” responses.

We also assessed the palatability and physical characteristics of IFAS and MMS supplements across four domains: taste, smell, appearance, and ease of swallowing. As shown in Table 4, participants rated IFAS significantly more favorably than MMS for its smell (82% vs. 63%). However, no statistically significant differences were found between IFAS and MMS in terms of taste, ease of swallowing, or appearance. These quantitative findings align with qualitative feedback from FGDs, where several participants described MMS as having a strong odor and a bitter, medicine‐like taste, especially when consumed on an empty stomach. One key insight from the FGDs was that many girls preferred to take their supplements after eating, a preference reflected in the endline survey results, where 67% of IFAS recipients and 79% of MMS recipients reported a preference for taking supplements post‐meal.

**Table 4:** Palatability and ease of consumption of IFAS and MMS (agree and strongly agree responses only).

Acceptability Indicator	IFAS (%) (*n*)	MMS (%) (*n*)	p‐value¹
Tastes good	78% (109)	73% (100)	0.36
Smells good	81% (113)	63% (86)	< 0.001
Easy to swallow	90% (126)	88% (120)	0.54
Looks good	94% (132)	92% (126)	0.61

¹*p‐values calculated using chi‐square test of independence to compare palatability and ease of supplement consumption between IFAS and MMS arms*. Percentages are based on available responses; totals are less than the full sample due to missing or “don't know” responses.

Finally, while both groups reported perceived health benefits and tended to prefer the regimen to which they were assigned, some participants described the once‐weekly IFAS regimen as less burdensome than the daily MMS routine. Participants attributed this difference to the ease of remembering the regimen and the teacher‐supported structure of school‐based supplementation, which is discussed in more detail under the interpersonal influences (Section 3.3).

### 3.3 Interpersonal Level: Social Acceptability and Support

We also explored how family and peer dynamics influence supplement adherence. Some girls found taking IFAS once a week at school easier to remember and less burdensome, due to the support provided by teachers and peers. However, some girls in the MMS group appreciated the daily routine, as it helped them feel consistent, responsible for their health, and cultivate beneficial habits. Some teachers noted that some girls struggled with daily MMS consumption. One teacher shared:

The girls liked to take it (IFAS) once a week. MMS had a lot of pills and that seemed tiring for them.

(Teacher, Participant 2, School 1 Teacher Discussion; Interpersonal level: perceived burden and teacher perspective)

Several themes emerged from the FGDs regarding adherence and challenges in consuming the supplements. Forgetfulness and the burden of maintaining a daily routine were identified as challenges to adherence and consistent consumption in the MMS group. Some participants expressed the view that taking supplements weekly at school would be easier than remembering to take them daily at home. However, this reflects opinion rather than direct experience, as participants could not switch regimens. One participant shared:

I prefer to take the once‐a‐week tablets (IFAS), because they're very good and easier to remember. As it's once a week, it's easier for me and I don't have to worry about taking them every day.

(IFAS supplement recipient, Participant 3, School FGD; Intrapersonal level: perceived burden)

Some participants in the MMS arm adopted strategies to support adherence, such as setting alarms or receiving reminders from their mothers. Although the MMS tracking sheets collected during the endline survey showed strong adherence, with over 95% of expected dates checked off from May to August, during the FGDs, participants did not mention this tool as a helpful strategy to support adherence.

### 3.4 Community Level: Cultural and Gendered Perceptions

A prominent challenge to acceptability at the community level was the presence of gendered misconceptions surrounding the supplements. Although sensitization activities and resources were provided at the start of the study to clarify that the supplements were not intended as birth control, several girls reported experiencing teasing, judgment, and misinformation from male peers who perceived the supplements as a form of birth control. Girls in the MMS arm also noted that the image of a pregnant woman on the label reinforced the misconception that the girls must be pregnant. One participant shared:

The boys never helped us. They just laughed at us and said stupid things, like that the pills we were taking were for not making children (birth control). This was very uncomfortable and disrespectful. They didn't understand the importance of what we were learning.

(MMS supplement recipient, Participant 10, School FGD; Community level: perceived norms and stigma)

Teachers at all three schools noted boys felt excluded from the program and expressed a desire to participate in the clubs, with one teacher sharing:

We didn't have any boys (in the program)…they just complained, like, ‘What about us? Why does the project only come to girls? Don't we have anemia?’

(Teacher, Participant 1, Teacher Discussion; Community level: program exclusion and gender norms)

In the endline survey, participants were asked whether anyone in their lives made it more difficult for them to take supplements. The majority in both arms reported that no one hindered their supplement use, though this response was significantly more common in the IFAS arm (87%) compared to the MMS arm (75%, *p* < 0.01). Parental encouragement was nearly universal, with over 97% of girls in both groups reporting that their parents supported supplement use. In contrast, social dynamics at school presented more challenges: 58% of girls in the MMS arm and 51% in the IFAS arm noted that boys spread rumors about MMS supplements and did not understand their intended purpose. Teachers also noted negative connotations and misconceptions surrounding the supplements. One teacher shared:

In the case of MMS, we didn't like the label on the bottle because it had a pregnant woman on it (and) it created a lot of controversy in the community because the interpretation…(is) that the girl who takes the supplement will never make children again.

(Teacher, Participant 3, School 1 Teacher Discussion; Community level: perceived norms and stigma)

### 3.5 Institutional Level: Feasibility and Delivery Challenges

At the institutional level, we examined the school environment, club structure, and engagement and satisfaction of both participants and teachers with the intervention. As shown in Table 5, most participants in both the IFAS and MMS arms rated the nutrition curriculum and club teachers positively. As shown in Table 5, over 90% of girls in each group rated the curriculum as excellent or good, with no statistically significant difference between groups (*p* = 0.06). Similarly, nearly all participants rated their teacher as excellent or good, with no significant difference between IFAS and MMS arms (*p* = 0.24).

**Table 5:** Participant ratings of the nutrition curriculum and teacher facilitation by supplement arm.

Question	Rating	IFAS (%) (*n*)	MMS (%) (*n*)	p‐value¹
Nutrition curriculum				0.06
	Excellent	43% (60)	36% (49)	
	Good	48% (67)	58% (80)	
	Okay	3% (4)	0% (0)	
	Not very good	6% (9)	6% (8)	
Teacher				0.24
	Excellent	39% (55)	33% (45)	
	Good	54% (76)	62% (85)	
	Okay	1% (2)	0% (0)	
	Not very good	5% (7)	5% (7)	

¹p‐values calculated using Fisher's exact test to assess differences in curriculum and teacher ratings between IFAS and MMS groups, due to small cell sizes. Percentages are based on available responses; totals are less than the full sample due to missing or “don't know” responses.

Several insights emerged from the qualitative inquiry. Both teachers and girls expressed positive affective attitudes (enthusiasm) toward the club format and nutrition curriculum. There was strong appreciation for the cooking demonstrations and menu creation activities, which teachers felt reinforced theoretical concepts. Some girls suggested that these demonstrations and recipes be shared with the broader community, especially their mothers and grandmothers. FGD participants also noted increased nutrition knowledge and reported changes in behavior, especially using locally sourced, affordable, iron‐rich foods when preparing meals. This, they said, helped them feel stronger and healthier. One participant commented:

I didn't know how to cook cassava leaf *matapa* (a traditional Mozambican dish typically prepared with ground peanuts) with peanuts, or how to make pumpkin jam, but now I know because I learned it here at school.

(MMS supplement recipient, Participant 7, School 1 FGD; Intrapersonal level: knowledge and perceived effectiveness)

A teacher shared similar sentiments:

I also liked talking about nutrition because our community is rich in diverse foods but lacks basic information on how our food can be consumed, but thanks to this project, our girls already have information and are informing their families that everything we have and produce is valuable.

(Teacher, Participant 3, School 1 Teacher Discussion; Institutional level: curriculum facilitation)

The FGDs also revealed several logistical and structural challenges. The club sessions met only once a week for 1 h, and both girls and teachers noted that extending the sessions to 2 h would allow for deeper engagement and learning. Some girls mentioned feeling hungry during the sessions, which impacted their ability to concentrate, and they recommended that snacks be provided in the future. Some teachers expressed difficulty balancing their roles and overlapping responsibilities as teachers and club facilitators, highlighting challenges in integrating the program into the school schedule. One teacher shared:

What I didn't like was the scheduling of the mentor (club leader) because I think we should schedule or program a day when all the leaders are present; it could be a Saturday.

(Teacher, Participant 3, School 3 Teacher Discussion; Institutional level: feasibility and implementation burden)

## 4 Discussion

Our study showed that both supplementation interventions, complemented by a school‐based nutrition education curriculum, were generally acceptable in our rural Mozambique context. To our knowledge, this is among the first studies to compare the acceptability of two distinct micronutrient supplementation strategies—school‐based weekly IFAS and home‐based daily MMS—among adolescent girls in a low‐resource setting.

While IFAS was significantly more acceptable than MMS in terms of palatability—including smell, appearance, and ease of swallowing—both arms reported improved overall well‐being, including improved energy, appetite, and relief from menstrual symptoms. These findings suggest that acceptability extends beyond taste and form, encompassing affective attitude and perceived effectiveness.

Regarding a sense of improved overall well‐being, similar findings have been reported in other adolescent studies, where micronutrient supplementation to treat anemia led to perceived improvements in fatigue and menstruation‐related weakness (Cooke et al. 2017), and increased overall energy and overall well‐being (Cliffer et al. 2023). While we did not find evidence in the literature indicating that MMS smelled worse than IFAS, the Vitamin B complex, a nutrient within MMS, is often associated with a bitter taste and a strong odor (Drewnowski and Gomez‐Carneros 2000). We hypothesize that MMS was perceived to have a stronger smell than IFAS due to its packaging. MMS was dispensed from bottles, exposing girls to the concentrated smell daily, while IFAS was handed out once a week, minimizing odor exposure.

Despite IFAS being more palatable than MMS, participants generally expressed a preference for the supplement regimen they were assigned to, suggesting a familiarity bias(Albarracín and Wyer 2000). Although each participant received only one formulation within their club, many observed their peers in parallel clubs receiving the alternate formulation. They may have formed perceptions through direct observation and informal feedback exchanged with friends both within and outside the school setting. Future studies could assess the influence of familiarity bias by employing a crossover design, where participants switch supplement regimens midway through the study period.

In our study, MMS acceptability was challenged by cultural and social factors, particularly community‐level miscommunication and gendered misconceptions. Even girls receiving weekly IFAS—which was not distributed in a pregnancy‐labeled bottle—also reported teasing. These findings align with broader literature documenting how adolescent girls' health interventions perceived to be linked to sexuality or reproduction can become stigmatized within school environments, particularly where gender norms and limited sexual and reproductive health literacy shape peer interpretations (Gillespie et al. 2022). Despite these social challenges overall engagement with the intervention did not appear diminished, as participants rated the nutrition curriculum and teachers' facilitation positively, with no significant differences between groups.

Given these misconceptions, one strategy is to apply adolescent‐centered design approaches to redesign the MMS label in a way that reduces ambiguity and misconceptions. and that reflects the preferences of the girls and their interpretations by the boys. Human‐centered design approaches have been successfully used globally, including in East Africa, to increase product desirability, acceptability, and uptake among youth (Wilson et al. 2022) (Mukherjee et al. 2022). A similar approach could be applied for non‐pregnant adolescent girls.

Another strategy to overcome misconceptions regarding supplements is to include boys in the intervention. Although anemia‐related DALYs are higher among girls than boys (Safiri et al. 2021), and the anemia prevalence rates are unacceptably high among girls in Monapo District, it is likely that the anemia prevalence rates are also unacceptably high for boys. Weekly IFAS programs typically target girls (WHO 2018), but gender‐inclusive nutrition and anemia prevention programs may normalize supplementation, reduce misunderstanding, and encourage peer support for adolescent health and nutrition. Discussions with the Monapo Head of the Department of Education revealed that boys were largely excluded from interventions, as non‐government organizations (NGOs) focused primarily on girls to address historical gender disparities (F. Mussa, personal communication, February 19 2024). However, there is growing recognition that including boys fosters gender equitable outcomes, promotes mutual respect, and creates opportunities for all genders (UN Women 2025). Future studies should evaluate the impact of including boys on the acceptability and effectiveness of supplementation programs, as more gender equitable strategies could lead to greater comprehensive impact.

Although teachers reported strong engagement with delivering both the curriculum and supplementation activities, reports of scheduling challenges and competing responsibilities raise important questions about long‐term sustainability. The stipend provided during the study period may not be feasible under routine programmatic conditions, and facilitating clubs alongside supplement distribution represents an opportunity cost within already constrained school systems. Without integration into existing school health structures or adjustments to reduce teacher burden, reliance on teacher‐led implementation may limit scalability. Future implementation research should examine workload distribution, cost implications, and potential adaptations, such as integration into standard curricula or alternative facilitators, to enhance sustainability.

Our emergent model (Figure 1) is derived from the study findings organized across four levels of the SEM—intrapersonal, interpersonal, community, and institutional—to illustrate the multifaceted influences on adolescent girls' acceptability of micronutrient supplementation. By overlaying constructs from the TFA onto each SEM level, the model highlights the pathways through which individual, relational, and structural factors intersect to influence adolescent engagement with supplementation. For example, reported increases in energy and appetite reflect the TFA construct of perceived effectiveness at the intrapersonal level, while girls' descriptions of difficulty maintaining daily routines align with the construct of burden. Peer teasing illustrates interpersonal influences on social acceptability, whereas labeling‐related stigma reflects broader community‐level norms and interpretations of supplementation. Parental reminders and teacher supervision represent interpersonal support mechanisms. Institutional‐level factors, such as scheduling constraints and teacher workload, correspond to feasibility and perceived burden within the implementation context. This visual and conceptual tool aims to inform adolescent health programming and policy by emphasizing the need for gender‐sensitive, socially aware, and contextually grounded interventions.

Our study findings may be applicable to adolescent girls who are not in school. Mozambique has one of the highest gender inequality indices in the world (United Nations Development Programme n.d.), with more than half of girls dropping out of school before fifth grade (USAID Advancing Girls' Education 2023). Out‐of‐school adolescents are more at risk for micronutrient deficiencies, as they miss out on school environments that often provide school meals and supplementation initiatives (UNICEF n.d.). Weekly school‐based IFAS may also be difficult to implement in contexts with high rates of political insecurity and gender‐based violence, which limit the mobility of adolescent girls and contribute to school absenteeism. Hence, the home‐based MMS delivery model described in this paper may offer a more acceptable and feasible solution for addressing high anemia rates among adolescent girls in such contexts, compared to requiring them to meet weekly to receive IFAS. Home‐based IFAS delivery models could also be explored. Future studies should explore supplementation strategies targeting out‐of‐school girls to promote equity and contribute to reducing national anemia prevalence.

### 4.1 Limitations

Our study has several limitations. First, social desirability bias may have influenced participants' reports of high acceptability for the supplements and the nutrition curriculum. To address this, the purpose of the endline survey and FGDs was clearly explained during the consent process, and participants were encouraged to provide honest responses. Additionally, the curriculum encouraged openness and camaraderie to foster a sense of safety and belonging within each club. Second, familiarity bias could have influenced participants' supplement preferences, as each participant was exposed to only one regimen. Third, while we collected MMS tracking sheets to monitor adherence, a more robust measure of adherence, such as pill counts to determine the number of supplements remaining in each bottle, would have provided a clearer picture of compliance. Although club attendance appeared high, attendance records were not systematically retained for analysis, and political unrest surrounding the Mozambique elections disrupted school activities near the endline, limiting our ability to track attendance consistently. Future studies should incorporate more systematic monitoring of both adherence and participation. Fourth, data collection occurred during a period of political instability surrounding the Mozambique presidential election in October 2024. Some teachers temporarily stepped away from their teaching responsibilities to support election‐related activities, which reduced participation in the teacher discussion groups and may have limited the range of teacher perspectives captured in the qualitative findings. Fifth, while teachers were the unit of randomization, we did not collect detailed quantitative measures of school‐level characteristics (e.g., infrastructure, distance, teacher workload capacity) that may influence implementation and acceptability. Unmeasured school‐level contextual differences could therefore have contributed to variation in reported experiences. Finally, although menarche status differed slightly between arms at baseline and may have shaped perceived effectiveness, the majority of girls in both arms had reached menarche, and overall acceptability ratings were high in both groups, and therefore unlikely to have meaningfully altered overall acceptability patterns.

### 4.2 Strengths

Our study has many strengths. The cluster‐randomized trial design helped ensure comparability in baseline demographic characteristics between the study arms. We assessed the acceptability of school‐based curriculum and IFAS and home‐based MMS delivery models in a real‐world, resource‐constrained, and fragile context, generating findings that can inform adolescent nutrition policy and programming in Mozambique. By applying a mixed‐methods process evaluation to evaluate acceptability, we were able triangulate and validate our findings across different methods and perspectives (Denzin 2006). Additionally, we applied a gender and equity lens, allowing us to explore structural and social barriers, such as negative perceptions, misconceptions, and gendered norms, that influence supplement acceptability and adherence.

## 5 Conclusions

Our study found that both school‐based iron and folic acid supplementation (IFAS) and home‐based multiple micronutrient supplementation (MMS), when complemented by a school‐based nutrition education curriculum, were acceptable among adolescent girls in rural Mozambique. Participants reported positive affective attitudes toward both the supplementation strategies and the nutrition curriculum, recognizing their value and expressing motivation to participate. However, the daily MMS regimen was associated with greater perceived burden, and its acceptability was influenced by palatability concerns and labeling‐related gendered misconceptions. Redesigning the bottle label for clarity and engaging male adolescents may help address these social challenges. Home‐based supplementation may offer a viable strategy for reaching out‐of‐school girls in fragile contexts. Future programs should integrate user‐centered design, address gender norms, and focus on reaching out‐of‐school girls to scale equitable and acceptable micronutrient supplementation models in similar settings.

## Author Contributions

S.B. designed the study and study instruments, led the qualitative and quantitative analysis, interpretation of the data, and drafted the manuscript. L.M., M.M., and T.B. contributed significantly to the implementation of the study activities. J.W. coded the F.G.D. and group meeting transcripts, and T.K. and J.W. helped with the quantitative analysis. C.M.G., E.L., J.G., J.P., C.T., T.D., A.D.T., and A.S.D. provided technical input on all aspects of the study and commented on subsequent drafts. All authors read and approved the final manuscript.

## Conflicts of Interest

The authors declare no conflicts of interest.

## Data Availability

The data that support the findings of this study are available from the corresponding author upon reasonable request.
